# Rapid evaluation of seed vigor by the absolute content of protein in seed within the same crop

**DOI:** 10.1038/s41598-018-23909-y

**Published:** 2018-04-03

**Authors:** Daxing Wen, Hongcun Hou, Aiju Meng, Jie Meng, Liuyong Xie, Chunqing Zhang

**Affiliations:** 1 State Key Laboratory of Crop Biology, Shandong Agricultural University, Tai’an, Shandong Province 271018 P. R. China; 2 College of Agronomy, Xinjiang Agricultural University, Urumqi, Xinjiang 830052 P. R. China

## Abstract

Seed vigor, an important index of seed quality, determines the potential for rapid and uniform emergence of plants. The objective of this study was to explore a rapid method for evaluating seed vigor. To analyze the correlation of seed traits and seedling traits related to seed vigor, we designed five experiments including nitrogen fertilizer, irrigation and seed sorting treatments in wheat. The results showed that only the absolute content of protein (ACP) in wheat seed was significantly correlated with plant dry weight in five experiments. Subsequently, another experiment including 30 wheat seed lots was used to validate the above results. Although 100-grain weight was also correlated with plant dry weight (R = 0.799, p < 0.01), the correlation coefficient was lower than that between ACP in seed and plant dry weight (R = 0.897, p < 0.01). Moreover, the results of three experiments using maize seeds was similar with above. The relative content of protein in seed detected by near-infrared spectrum combining with seed weight could realize rapid and nondestructive testing ACP in seed. Collectively, ACP in crop seed could be applied in rapid evaluation of seed vigor and could potentially be used for processing and screening high vigor seeds.

## Introduction

Seed vigor determines the potential for rapid and uniform emergence of plants under a wide range of field conditions^1,2^. Seedling vigor mainly reflects seedling weight or height, which usually neglects germination speed^3,4^. Moreover, most experimental conditions involved in seedling vigor in these studies (germination time, temperature, management, etc.) were different with standard germination testing in seed industry. Nowadays, seed industry uses seed vigor to evaluate seed quality. High seed vigor is associated with the potential for increasing growth and productivity in agricultural production^5^. However, standard testing of seed vigor needs one or more weeks, which is labor intensive and time consuming. Therefore, there is tremendous interest in and demand for exploring rapid testing methods for seed vigor.

Seed weight and seed nutrient content affect plant growth at seedling stage^6,7^. Previous studies show a close relationship between seed size or weight and early vigor in rice^3,8^. In general, rice seed with thin hull and large embryo are desirable for seed vigor^8^. Seed weight was correlated with plant growth at low P supply up to the flowering stage in soybean, and the effect of seed weight at low P conditions was larger than that at high P conditions^6^. Seedling vigor was remarkably related to seed size and protein concentration, but was most closely correlated with protein content per seed in wheat^7,9^. Seed mass and total oil + protein calorie content of the individual quiescent cotton seed was strongly associated with early seedling vigor in upland cotton^10^.

Seed storage nutrients (carbohydrates, lipids, proteins, etc.) are crucial for seed vigor, but which one trait can be used to evaluate seed vigor needs further study. Seed carbohydrate reserve is important for rice seed vigor because that breakdown of the starch stored in the endosperm provides carbohydrates for germination and heterotrophic growth^11^. Seed protein content is known to vary considerably affecting by species, genotype and environment. The effect of seed protein content on seedling vigor has been studied in many species, such as wheat, barley and oats^4,12–14^. Nitrogen mobilization from storage protein is required to meet the amino acid demands at the early stage of germination^15^. In low fertility conditions, seedling vigor are strongly influenced by nitrogen from seed reserves in soybean^16^. When seed need to be deep seeding under low soil moisture, a cost-effective method for increasing wheat seedling vigor is increasing seed protein content by nitrogen fertilization of mother plants^17^. Changes of protein concentration in rice seed had no effect on germination rate and energy, which might be due to the decrease in protein content at elevated carbon dioxide concentration (CO_2_) did not exceed a threshold value^18^. For many crops, seed contain sufficient P to sustain optimal growth of seedlings for several weeks after germination^19^.

In this study, we found that the absolute content of protein in wheat seed and maize seed was significantly correlated with plant dry weight at seedling stage. The objective of this study was to explore if the absolute content of protein in seed could be applied in rapid evaluation of seed vigor. In addition, this method could potentially be used for processing and screening high vigor seeds.

## Results

### Correlation analysis of plant dry weight and wheat seed traits

**Figure 1:**
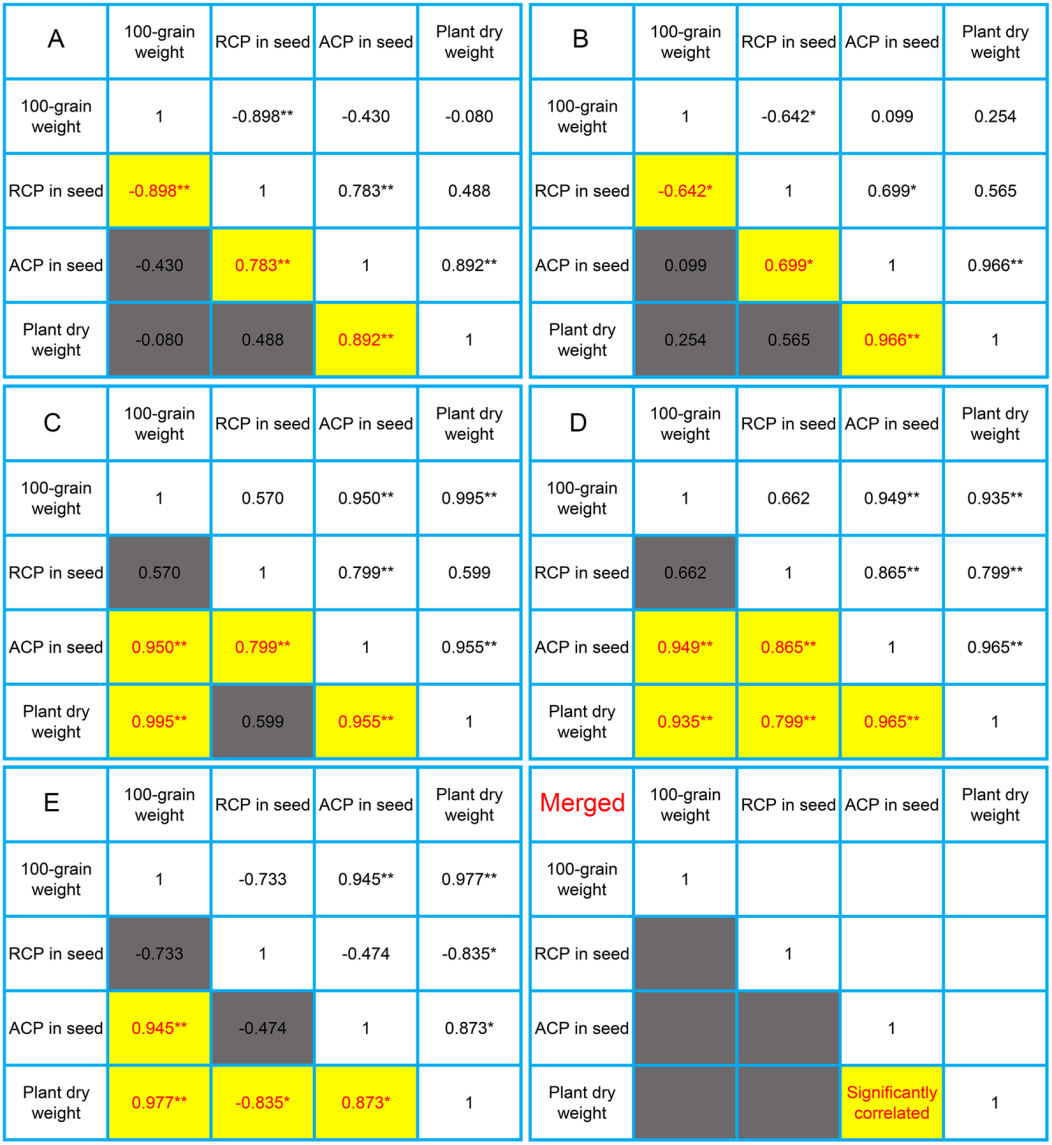
Correlation analysis of seed traits and seedling traits in wheat. (**A**) Wheat seed produced under different nitrogen level. Four nitrogen level: N0 (0 kg/ha), N168 (168 kg/ha), N240 (240 kg/ha, the usual nitrogen fertilizer level for winter wheat production in the North China Plain) and N300 (300 kg/ha). (**B**) Wheat seed came from big spike of experiment A, and other treatments of experiment B was the same with experiment A. (**C** and **D**) Wheat seed came from different parts of spike (top 1/4, middle 1/3 and bottom 1/4) under N0 and N240 treatments, respectively. (**E**) Wheat seed produced under different irrigation after anthesis. Two water level: normal irrigation and no irrigation with control rainfall. RCP: the relative content of protein (%). ACP: the absolute content of protein (mg seed^−1^). Asterisks denote a significant correlation (*p < 0.05; **p < 0.01).

In our previous study, we found that protein metabolism and nitrogen metabolism play an important role in seedling establishment and seed vigor. To study if there is a seed trait can be used to evaluate plant dry weight and seed vigor, we designed a series of experiments. In each experiment, we first tested 100-grain weight and the relative content of protein (RCP, %) in seed and then calculate the absolute content of protein (ACP, mg seed^−1^) in wheat seed. Moreover, we used standard germination test to detect plant dry weight and seed vigor. In experiment A, wheat seed produced under different nitrogen level (four nitrogen level: 0 kg/ha, 168 kg/ha, 240 kg/ha (the usual nitrogen fertilizer level for winter wheat production in the North China Plain) and 300 kg/ha). Correlation analysis showed that RCP in wheat seed was significantly correlated with 100-grain weight and ACP in wheat seed, and plant dry weight was remarkably correlated with ACP in wheat seed (Fig. 1A). Wheat seeds coming from big spike of experiment A were used in experiment B, and correlation analysis result was consistent with that in experiment A (Fig. 1B). Wheat seeds in experiment C and D came from different parts of spike (top 1/4, middle 1/3 and bottom 1/4) under N0 and N240 treatments, respectively. Among these seeds, there were significantly different in plant dry weight and seed vigor. Correlation analysis of experiment C and D showed that there was no significant correlation between 100-grain weight and RCP in wheat seed (Fig. 1C,D). Wheat seed produced under different irrigation after anthesis (two water level: normal irrigation and no irrigation with control rainfall) was used in experiment E. The results showed that there was no significant correlation between RCP in wheat seed and ACP in wheat seed. Therefore, after merged results of the five experiments, we found that only ACP in wheat seed was significantly correlated with plant dry weight.

### ACP in wheat seed can be used to evaluate plant dry weight and seed vigor

To study whether ACP in wheat seed can be used to evaluate plant dry weight and seed vigor, we used 30 wheat seed lots (including 27 wheat cultivars seeds produced in normal regional trial, two wheat cultivars seeds produced under drought condition and one wheat cultivars seeds produced under low nitrogen condition) stored about 6 months at room temperature to validate the above results. Correlation analysis showed that ACP in wheat seed was significantly correlated with plant dry weight (R = 0.897, Table 1). Although 100-grain weight was also significantly correlated with plant dry weight (R = 0.799), the correlation coefficient was lower than that between ACP in wheat seed and plant dry weight. Moreover, the correlation coefficient between ACP in wheat seed and shoot dry weight (R = 0.749) or root dry weight (R = 0.749) was lower than that between ACP in wheat seed and plant dry weight (R = 0.897) which was the biggest one in Table 1. Therefore, it would be better to use ACP in wheat seed to predict plant dry weight. In addition, ACP in wheat seed could also be applied in evaluating vigor of seed which was newly harvested or short time storage due to little difference in germination index. ACP in wheat seed could potentially be used for processing and screening high vigor seeds.

**Table 1:** Correlation analysis between seeds and plants by using 30 wheat seed lots.

	100-grain weight	RCP in wheat seed	ACP in wheat seed	Plant dry weight	Shoot dry weight	Root dry weight
100-grain weight	1	−0.272	0.837**	0.799**	0.602**	0.742**
RCP in wheat seed	−0.272	1	0.291	0.166	0.272	−0.014
ACP in wheat seed	0.837**	0.291	1	0.897**	0.749**	0.749**
Plant dry weight	0.799**	0.166	0.897**	1	0.857**	0.810**
Shoot dry weight	0.602**	0.272	0.749**	0.857**	1	0.393*
Root dry weight	0.742**	−0.014	0.749**	0.810**	0.393*	1

RCP: the relative content of protein (%). ACP: the absolute content of protein (mg seed^−1^). Asterisks denote a significant correlation (*p < 0.05; **p < 0.01).

### Regression analysis of ACP in wheat seed and plant dry weight

**Figure 2:**
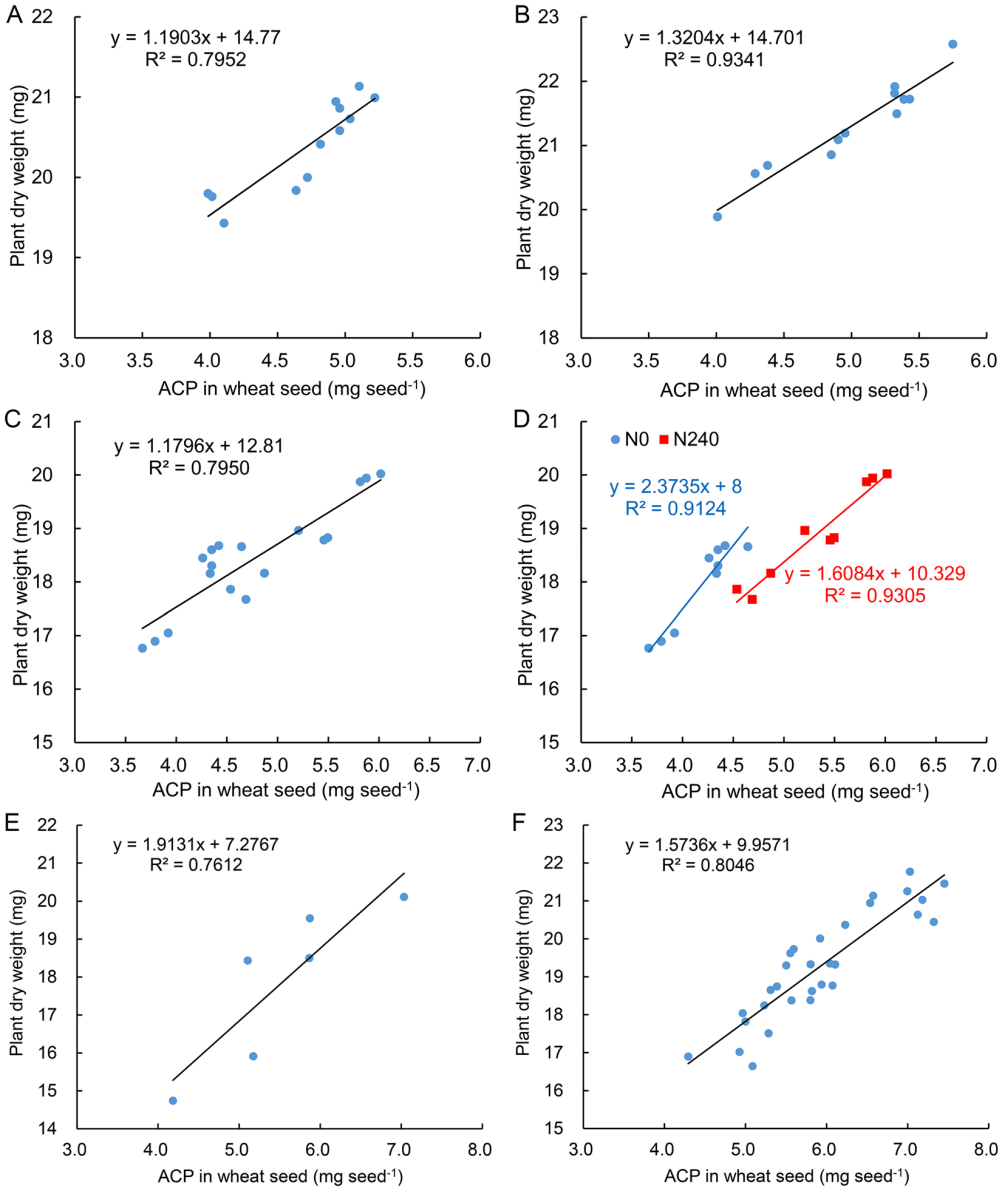
Regression analysis of ACP in wheat seed and plant dry weight at seedling stage. (**A**) Wheat seed produced under different nitrogen level. Four nitrogen level: N0 (0 kg/ha), N168 (168 kg/ha), N240 (240 kg/ha, the usual nitrogen fertilizer level for winter wheat production in the North China Plain) and N300 (300 kg/ha). (**B**) Wheat seed came from big spike of experiment A. (**C** and **D**) Wheat seed came from different parts of spike (top 1/4, middle 1/3 and bottom 1/4) under N0 and N240 treatments, respectively. (**E**) Wheat seed produced under different irrigation after anthesis. Two water level: normal irrigation and no irrigation with control rainfall. (**F**) Thirty wheat seed lots included 27 wheat cultivars seeds produced in normal regional trial, two wheat cultivars seeds produced in drought condition and one wheat cultivars seeds produced in low nitrogen condition. ACP: the absolute content of protein (mg seed^−1^).

We used the above six experiments to further analyzed regression analysis of ACP in wheat seed and plant dry weight. The regression equation was different among six experiments, but the overall trend was similar (R^2^ range: 0.7612–0.9341, Fig. 2A–F). Degree of fitting analyzed by using seeds of N0 and N240 (Fig. 2C) was similar with that analyzed by using only N0 seeds or only N240 seeds (Fig. 2D). Regression analysis of validated experiment using 30 wheat seed lots showed that degree of fitting was high (R^2^ = 0.8046, Fig. 2F).

### Correlation analysis of plant dry weight and maize seed traits

**Figure 3:**
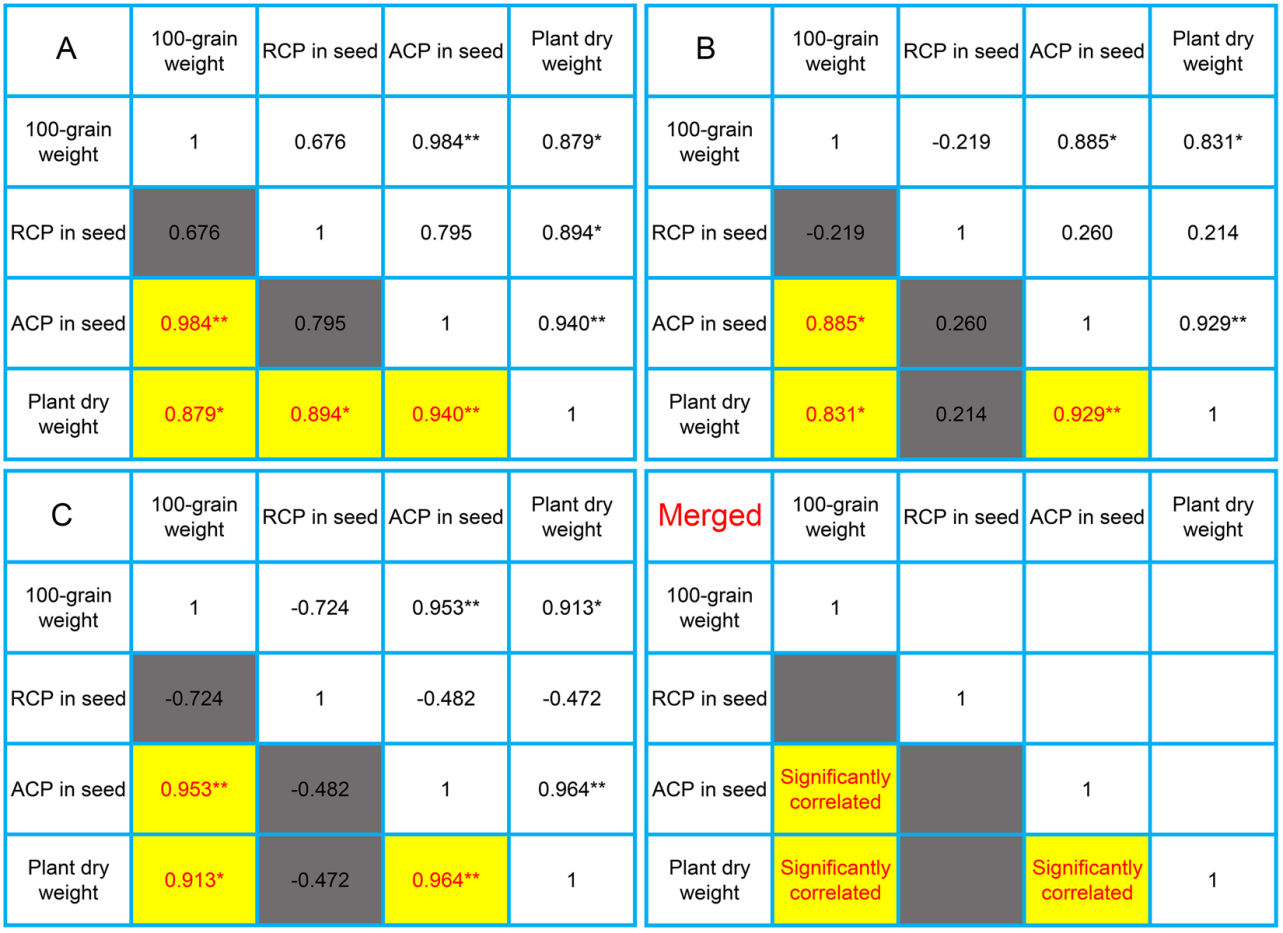
Correlation analysis of seed traits and seedling traits in maize. (**A**) Maize seed came from different parts of ear (top 1/4, middle 1/3 and bottom 1/4) of maize inbred line Z58. (**B**) Maize seed came from different parts of ear (top 1/4, middle 1/3 and bottom 1/4) of maize cultivars ZD958. (**C**) Maize seed came from the middle part of ear in two maize inbred lines (Z58 and C7-2) and one maize cultivar (ZD958). All maize seed produced under two water level, i.e. normal irrigation and no irrigation with control rainfall. RCP: the relative content of protein (%). ACP: the absolute content of protein (mg seed^−1^). Asterisks denote a significant correlation (*p < 0.05; **p < 0.01).

To validate whether ACP in maize seed can be used to evaluate plant dry weight, we designed three experiments (MA, MB and MC). In each experiments, we used maize seed produced under normal irrigation and drought stress after anthesis. We first tested 100-grain weight and RCP in maize seed and then calculate ACP in maize seed. Moreover, we used standard germination test to detect plant dry weight and seed vigor. Maize seeds in experiment MA came from different parts of ear (top 1/4, middle 1/3 and bottom 1/4) of maize inbred line Z58. Plant dry weight and seed vigor was also significantly different among seed of different parts of maize ear. Correlation analysis showed that plant dry weight was significantly correlated with 100-grain weight, RCP in maize seed and ACP in maize seed, and 100-grain weight was remarkably correlated with ACP in maize seed (Fig. 3A). In experiment MB, maize seed came from different parts of ear (top 1/4, middle 1/3 and bottom 1/4) of maize cultivar ZD958. The results was similar with that of experiment MA, but there was no significant correlation between plant dry weight and RCP in maize seed (Fig. 3B). In experiment MC, we used two maize inbred lines (Z58 and C7-2) and one maize cultivar (ZD958) as materials, and seed came from the middle part of ear. The results were the same as that in experiment MB except different correlation coefficient (Fig. 3C). Comprehensive analysis of the above three experiments, plant dry weight was significantly correlated with 100-grain weight and ACP in maize seed, and100-grain weight was remarkably correlated with ACP in maize seed. Although plant dry weight was significantly correlated with 100-grain weight, the correlation coefficient was lower than that between plant dry weight and ACP in maize seed (R range: 0.929–0.964) in the above three experiments. Therefore, it would be better to use ACP in maize seed evaluating plant dry weight. Correlation coefficient between plant dry weight and ACP in maize seed was higher than that in wheat seed. Moreover, ACP in maize seed could also be used for evaluating vigor of seed which was newly harvested or short time storage due to little difference in germination index. ACP in maize seed could potentially be applied in processing and screening high vigor seeds.

### Regression analysis of ACP in maize seed and plant dry weight

**Figure 4:**
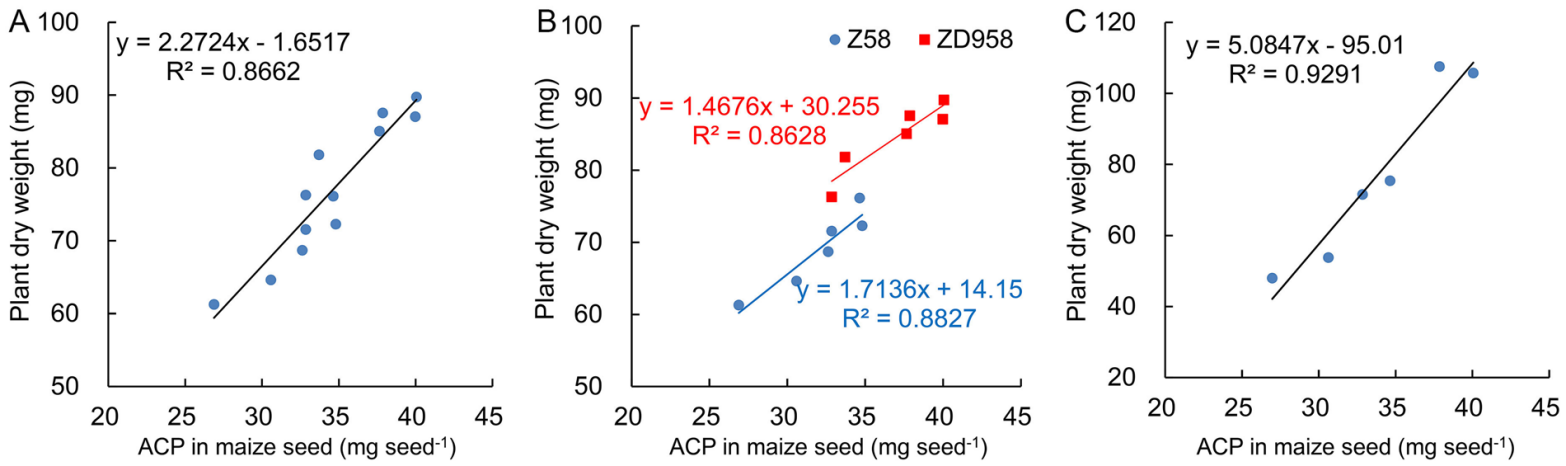
Regression analysis of ACP in maize seed and plant dry weight at seedling stage. (**A**) Regression analysis of ACP in maize seed and plant dry weight by using all seeds in experiments MA and MB. (**B**) Two regression analysis of ACP in maize seed and plant dry weight by using seeds in experiments MA and MB, respectively. (**C**) Regression analysis of ACP in maize seed and plant dry weight by using seeds in experiment MC. ACP: the absolute content of protein (mg seed^−1^).

We used the above three experiments to further analyzed regression analysis ACP in maize seed and plant dry weight. The regression equation was different among three experiments, but the overall trend was similar (R^2^ range: 0.8628–0.9291, Fig. 4). Degree of fitting analyzed by using Z58 and ZD958 seeds (Fig. 4A) was similar with that analyzed by using only Z58 seeds or only ZD958 seeds (Fig. 4B). Degree of fitting between plant dry weight and ACP in maize seed was higher than that in wheat seed.

## Discussion

Seed vigor can be evaluated by many methods, such as vigor index, accelerated aging test and cold test^20,21^. There was no universal seed vigor testing methods, due to seed vigor influenced by species, genotype and environment conditions. The most widely used methods are standard germination test and accelerated aging test. Seed vigor is more complex than seedling vigor^22^. In some cases, trends of seedling vigor are consistent with seed vigor. However, seedling vigor might be different with seed vigor in seed stored long time.

Although seed weight was correlate with seed vigor and seedling vigor in most cases, ACP in seed was more suitable for evaluating plant dry weight and seed vigor. Many studies have evaluated the relationship between seed weight and seedling growth among different species^23^. Large seed are usually assumed to be advantageous for seedling establishment in nutrient-limited conditions, but little consistent evidence to support this hypothesis^23^. Wheat seedling vigor was remarkably increased by seed weight and percent protein in growth chamber experiments, but most of the variation was attributed to the amount of nitrogen in the seed and not to seed weight or the percentage protein^24^. The results was similar with this study. Altogether, our results indicate that the correlation coefficient between ACP in wheat seed and plant dry weight was higher than that between 100-grain weight and plant dry weight.

Snider observed positive association between mean oil and protein kcal content per seed and seedling vigor in cotton^10^. Oil and protein are predominant storage nutrients in cotton seed, and which one is more important for seedling vigor is still unclear. Oil and protein of seed may be all limiting factor for seedling vigor in cotton. In wheat seed, starch and protein are main storage nutrients, and the total weight of starch and protein is approximate seed weight. In this study, we also found that seed storage protein played an important role in seedling growth and seed vigor. Starch might also affect seed vigor, but starch content in wheat seed is usually not too low compared with other storage nutrients. In general, starch content may be not limiting factor for seed vigor in wheat. Therefore, it would be better to use ACP in wheat seed evaluating plant dry weight or seed vigor.

Seed protein content usually means the relative content of protein (RCP, % or mg/g) in seed in previous studies^25^. Only few studies analyzed the absolute content of protein (ACP, mg seed^−1^) in seed influenced on seedling vigor^7^. When seed with high RCP in seed and low seed weight was used in experiment, there was no significant correlation between RCP in seed and plant dry weight. Collectively, ACP in seed was better than RCP in seed for evaluating plant dry weight and seed vigor.

Seed vigor prediction could decrease production risks associated with poor stand establishment or influence early season management decisions^10^. In previous studies, nitrogen and protein content of seed were usually determined by the microKjeldah1 procedure^12,13,24^. The method need grind seed and relative long testing time, which is difficult to meet rapid and nondestructive testing seed vigor. In this study, we used near-infrared spectrum to detect RCP in seed and then multiply RCP in seed by seed weight to calculate ACP in seed. ACP in seed could be used to predict plant dry weight and seed vigor.

In conclusion, the absolute content of protein in wheat seed and maize seed was significantly correlated with plant dry weight at seedling stage. RCP in crop seed detected by near-infrared spectrum combining with seed weight could realize rapid and nondestructive ACP in crop seed. Thus, ACP in crop seed could be applied in rapid evaluation of seed vigor and plant dry weight at seedling stage within the same crop. Moreover, ACP in crop seed could potentially be used to process and screen high vigor seeds.

## Materials and Methods

### Materials

Wheat seeds were produced in field trials in Dongwu Village (35°57′N and 117°3′E, Dawenkou Town, Tai’an City, Shandong Province, China) during the period of wheat growth (October–June) in 2014–2015 and 2015–2016. Wheat seeds came from several experiments with nitrogen fertilizer application treatments (2014–2015), irrigation treatments (2015–2016) and normal regional trial (2015–2016). We set four nitrogen fertilizer levels (0 kg/ha, 168 kg/ha, 240 kg/ha (the usual nitrogen fertilizer level for winter wheat production in the North China Plain) and 300 kg/ha in nitrogen fertilizer experiment. In other experiments, 240 kg/ha nitrogen fertilizer was applied. Phosphorus and potassium fertilizers were applied at rates of 120 kg/ha P_2_O_5_ and 75 kg/ha K_2_O, respectively. Base fertilizer was composed of 40% nitrogen fertilizer, 100% phosphorus fertilizer and 60% potassium fertilizer. At jointing stage, the residual 60% nitrogen fertilizer and 40% potassium fertilizer were used as topdressing. Irrigation, diseases, and insect pests were adequately controlled. Maize seeds were produced in the field trials during the period of maize growth (June–October) in 2015. Maize seeds came from several experiments with irrigation treatments. After anthesis, we set two water level, i.e. normal irrigation and no irrigation with control rainfall. Phosphorus and potassium fertilizers were applied before sowing at rates of 75 kg/ha P_2_O_5_ and 100 kg/ha K_2_O, respectively. Nitrogen fertilizer was applied as topdressing at jointing stage (60 kg/ha) and large-bell stage (60 kg/ha), respectively. The area has a semi-humid continental temperate monsoon climate. The soil type is sandy loam.

### Seed moisture content and 100-grain weight

Seed moisture content was detected with DA7200 (Perten, Stockholm, Sweden)^26^. Three replications of 100 seeds were randomly selected from each treatment for 100-grain weight measurement.

### Seed protein content assessment

The relative content of protein (RCP, %) in seed was detected with DA7200 (Perten, Stockholm, Sweden) based on near-infrared spectrum^27,28^. Multiply RCP by seed weight to calculate the absolute content of protein (ACP, mg seed^−1^) in seed.

### Evaluation of seedling and seed vigor

Standard germination tests were conducted according to the International Seed Testing Association with minor modifications^29^. Germination boxes (17 cm length, 11 cm width and 7 cm height) were used in this study. Sprouting bed was consisted of fine silica sand with a diameter of 0.05 to 0.8 mm. In per germination box, base sand was consisted of 4 cm height silica sand with 60% saturation moisture content. Randomly selected 100 wheat seeds (or 50 maize seeds) were sowed in the surface of base sand, and then the seed were covered with 2 cm height silica sand with 60% saturation moisture content. All germination boxes were placed in a growth chamber at 20 ± 1 °C for wheat (or 25 ± 1 °C for maize), 70% relative humidity, illumination conditions of 4000 lux and 24 h light photoperiod for eight days (wheat) or seven days (maize). At 3 days after imbibition (DAI), the lids of germination box were removed and then seedlings were sprayed with water about 20 mL/d from germination 4 to 8 DAI (wheat) or 7 DAI (maize). At germination 8 DAI (wheat) or 7 DAI (maize), seedlings were washed to remove silica sand, and then the remained seeds were removed from seedlings. Meanwhile, we record the number of seedlings per day that were used to calculate germination index (GI) and vigor index (VI). GI = ∑ (Gt/Dt), where Gt is the number of the germinated seed on day t and Dt is time corresponding to Gt in days. VI = GI × S, where S is plant dry weight.

### Measurements of seedling dry weight

Seedling dry weight was assessed by shoot dry weight, root dry weight and plant dry weight. Shoot and root were separated to measure dry weight. Shoot and root were heated in an air oven at 105 °C for 30 min, and then dried to constant weight at 80 °C. Plant dry weight is equal to shoot dry weight plus root dry weight.

### Statistical analysis

Correlation analysis and regression analysis were performed using SPSS 19.0 software (SPSS, Inc., Chicago, USA).

## References

[1] Rajjou L (2012). Seed Germination and Vigor. Annu. Rev. Plant Biol..

[2] Finch-Savage WE, Clay HA, Lynn JR, Morris K (2010). Towards a genetic understanding of seed vigour in small-seeded crops using natural variation in Brassica oleracea. Plant Sci..

[3] Lu X (2007). Genetic dissection of seedling and early vigor in a recombinant inbred line population of rice. Plant Sci..

[4] Lowe, L. B. & Ries, S. K. Effects of environment on the relation between seed protein and seedling vigor in wheat. *Can. J. Plant Sci.* **52**, 157–164 (1972).

[5] Han Z (2014). QTLs for Seed Vigor-Related Traits Identified in Maize Seeds Germinated under Artificial Aging Conditions. PLoS One.

[6] Vandamme E, Pypers P, Smolders E, Merckx R (2016). Seed weight affects shoot and root growth among and within soybean genotypes beyond the seedling stage: implications for low P tolerance screening. Plant Soil.

[7] Ries SK, Everson EH (1973). Protein content and seed size relationships with seedling vigor of wheat cultivars. Agron. J..

[8] Huang M (2017). Morphological and physiological traits of seeds and seedlings in two rice cultivars with contrasting early vigor. Plant Prod. Sci..

[9] Ries SK, Ayers G, Wert V, Everson EH (1976). Variation in protein, size and seedling vigor with position of seed in heads of winter wheat cultivars. Can. J. Plant Sci..

[10] Snider JL, Collins GD, Whitaker J, Chapman KD, Horn P (2016). The impact of seed size and chemical composition on seedling vigor, yield, and fiber quality of cotton in five production environments. Field Crop. Res..

[11] Cui K (2002). Molecular dissection of seedling-vigor and associated physiological traits in rice. Theor. Appl. Genet..

[12] Ayers GS, Wert VF, Ries SK (1976). The Relationship of Protein Fractions and Individual Proteins to Seedling Vigour in Wheat. Ann. Bot.-London.

[13] Schweizer CJ, Ries SK (1969). Protein Content of Seed: Increase Improves Growth and Yield. Science.

[14] Welch RW (1977). Seedling vigour and grain yield of cereals grown from seeds of varying protein contents. J. Agr. Sci..

[15] Soriano D (2011). Seed reserve composition in 19 tree species of a tropical deciduous forest in Mexico and its relationship to seed germination and seedling growth. Ann. Bot.-London.

[16] Naegle ER, Burton JW, Carter TE, Rufty TW (2005). Influence of seed nitrogen content on seedling growth and recovery from nitrogen stress. Plant Soil.

[17] Torres JL, Paulsen GM (1982). Increasing seed protein content enhances seedling emergence and vigor in wheat. J. Plant Nutr..

[18] Chen C (2015). Seed vigor of contrasting rice cultivars in response to elevated carbon dioxide. Field Crop. Res..

[19] White PJ, Veneklaas EJ (2012). Nature and nurture: the importance of seed phosphorus content. Plant Soil.

[20] Guan YJ (2013). Time series regression analysis between changes in kernel size and seed vigor during developmental stage of sh2 sweet corn (Zea mays L.)seeds. Sci. Hortic.-Amsterdam.

[21] Marcos-Filho J (2015). Seed vigor testing: an overview of the past, present and future perspective. Sci. Agr..

[22] Ellis RH (1992). Seed and seedling vigour in relation to crop growth and yield. Plant Growth Regul..

[23] Hanley ME, Cordier PK, May O, Kelly CK (2007). Seed size and seedling growth: differential response of Australian and British Fabaceae to nutrient limitation. New Phytol..

[24] Bulisani EA, Warner RL (1980). Seed Protein and Nitrogen Effects Upon Seedling Vigor in Wheat. Agron. J..

[25] Lowe LB, Ayers GS, Ries SK (1972). Relationship of Seed Protein and Amino Acid Composition to Seedling Vigor and Yield of Wheat1. Agron. J..

[26] Wen D (2017). A loose endosperm structure of wheat seed produced under low nitrogen level promotes early germination by accelerating water uptake. Sci. Rep.-UK.

[27] Delwiche SR (1998). Protein Content of Single Kernels of Wheat by Near-Infrared Reflectance Spectroscopy. J. Cereal Sci..

[28] Baye TM, Pearson TC, Settles AM (2006). Development of a calibration to predict maize seed composition using single kernel near infrared spectroscopy. J. Cereal Sci..

[29] ISTA (ed.) *International rules for seed testing*. (ISTA, Switzerland; 2010).

